# Intestinal Permeability and Depression—A Narrative Review of Selected Blood-Based Biomarkers

**DOI:** 10.3390/ijms262010076

**Published:** 2025-10-16

**Authors:** Anca C. Bibolar, Bianca D. Crecan-Suciu, Ramona L. Păunescu, Vlad-I. Nechita, Olivia Verisezan-Roșu, Ioana V. Micluția

**Affiliations:** 1 Department of Neurosciences, “Iuliu Hațieganu” University of Medicine and Pharmacy, 43 Victor Babeș Street, 400012 Cluj-Napoca, Romania; goron.anca03@gmail.com (A.C.B.); paunescu.ramona@umfcluj.ro (R.L.P.); imiclutia@umfcluj.ro (I.V.M.); 2 Department of Medical Informatics and Biostatistics, “Iuliu Hațieganu” University of Medicine and Pharmacy, 6 Pasteur Street, 400349 Cluj-Napoca, Romania; nechita.vlad@umfcluj.ro; 3 RoNeuro Institute for Neurological Research and Diagnostic, 400364 Cluj-Napoca, Romania; dr.olivia.rosu@gmail.com

**Keywords:** intestinal permeability, depression, zonulin, lipopolysaccharides (LPS), biomarkers

## Abstract

The intestinal barrier has recently gained attention as a contributor to the pathophysiology of depression. This narrative review examines the current literature on blood-based markers of intestinal permeability in patients with depression. A structured search of PubMed and EMBASE was performed. Both recent and older studies were included to capture key mechanisms and theoretical foundations. We focused on zonulin, intestinal fatty acid-binding protein (I-FABP), lipopolysaccharides (LPS), LPS-binding protein (LBP), and soluble CD14 (sCD14). While several studies report altered intestinal permeability markers in individuals with depression, results remain inconsistent. Factors such as small sample sizes and variability in measurement procedures complicate interpretation. In some cases, altered biomarker levels were associated with disease severity or response to antidepressant treatment, suggesting a potential role in patient stratification. However, current evidence does not support their routine use in clinical settings. Further research is needed to clarify their value in psychiatric populations. If validated, these markers may help identify inflammation-related depression subtypes and guide more precise treatment strategies.

## 1. Introduction

Depression is a widespread mental health disorder marked by persistent emotional and cognitive, as well as physiological disturbances [1]. While antidepressants such as selective serotonin reuptake inhibitors (SSRIs) and psychotherapy are the standard of care, roughly half of major depressive disorder (MDD) patients are poorly responsive [2]. Recent advances in neuroscience emphasize that depression arises from the interplay of several biological and environmental factors. Dysregulation of the hypothalamic–pituitary–adrenal (HPA) axis, persistent low-grade inflammation, impaired neuroplastic processes, and monoaminergic and endocannabinoid network imbalances are frequently discussed as potential key mechanisms contributing to the disorder. These proposed mechanisms have opened the way for innovative treatment options, ranging from neuromodulation techniques such as Transcranial Magnetic Stimulation (TMS) and vagus nerve stimulation (VNS), to rapid-acting antidepressants including esketamine, and even the controlled use of psychedelic agents. In parallel, digital tools and artificial intelligence are increasingly being explored both for diagnostic support and therapeutic monitoring. Moreover, growing evidence points to the gut–brain axis (GBA) as a promising frontier, with interventions such as probiotics and fecal microbiota transplantation (FMT) under investigation. These developments underscore the importance of adopting a comprehensive and integrative perspective on depression. However, standardized dysbiosis-related biomarkers remain unestablished.

Intestinal permeability biomarkers might aid in the evaluation of treatment-resistant depression, help stratify individuals based on gut-related inflammation, promote earlier identification of this specific subgroup of depressive patients and monitor treatment response. Given the emerging role of gut integrity in mental health, understanding the functional and structural basics of the intestinal barrier (IB) becomes essential. The human body is constantly exposed to various microorganisms and toxins. The IB captures nutrients and retains pathogenic microbes, chemicals, toxins or allergens. Structured in multiple layers, it acts as a physical and functional shield [3]. Tight junctions (zonula occludens -ZO), adherens junctions (zonula adherens), and desmosomes, reinforce the barrier at the epithelial level [4]. Compromising factors include psychological stress, dysbiosis, bacterial, parasitic or fungal infections, strenuous exercise, heat stress, alcohol, pesticides and antibiotics [5]. The integrity of the IB is often quantified by measuring intestinal permeability.

There is a scientific consensus regarding IB dysfunction in conditions where epithelial damage and local inflammation are evident, such as celiac disease, Crohn’s disease, and NSAID-induced ulceration. In these disorders, increased intestinal permeability is a well-established feature, often accompanied by structural abnormalities of the epithelial lining. The term “leaky gut”, while popularized in literature, represents a simplified description of this complex process. More recently, intestinal permeability has been studied in cases where overt inflammation or macroscopic damage may be absent. This condition is now associated with a wide range of disorders, including obesity, non-alcoholic fatty liver disease (NAFLD), non-alcoholic steatohepatitis (NASH), cirrhosis, cardiovascular diseases, type 1 diabetes, and various autoimmune conditions [6]. Studies also found subtle IB dysfunction in neuropsychiatric illnesses. Evidence indicates that both Parkinson’s disease (PD) and Alzheimer’s disease (AD) are associated with gut dysbiosis and increased intestinal permeability, suggesting a potential role of IB dysfunction in the pathophysiology of neurodegenerative disorders [7,8,9]. By simulating increased gut permeability through the administration of endotoxins, Behairi et al. (2016) created a mouse model characterized by memory impairment, increased systemic nitric oxide (NO) production, upregulated Aβ 1–42 expression in the brain, and neuronal degeneration resembling Alzheimer’s disease (AD) [10]. Autism and schizophrenia were also linked to “leaky gut” syndrome [11,12]. Intestinal permeability, as a relevant factor in depression, complements findings in other neuropsychiatric conditions and proposes a shared pathophysiological mechanism that involves GBA dysfunction. Modified levels of gut permeability markers in these conditions could suggest that milder, potentially subclinical, forms of IB dysfunction are sufficient to drive systemic immune activation, contributing to psychological distress. Increased intestinal permeability facilitates the translocation of microbial products, particularly bacterial endotoxins such as lipopolysaccharides (LPS), into the systemic circulation. Once in the bloodstream, these endotoxins interact with immune cells and activate immune pathways, leading to the release of pro-inflammatory cytokines (e.g., IL-1, IL-6, and TNF-α) and the establishment of a systemic inflammatory state. The sustained inflammatory milieu contributes to blood–brain barrier (BBB) dysfunction, thereby allowing peripheral inflammatory mediators to access the central nervous system and promote neuroinflammation [13,14,15]. The result is an impact on brain function via hypothalamic-pituitary-adrenal (HPA) axis stimulation, increased oxidative and nitrosative stress (O&NS) and microglial activation [16,17,18]. Moreover, LPS modify neurotransmitter balance, reducing 5-HT production [16]. Inflammation-related mechanisms will be further elaborated in the section specifically addressing the effects of LPS. The involvement of multiple physiological mechanisms underscores the potential relevance of intestinal permeability beyond the traditional boundaries of gastrointestinal medicine. This narrative review aims to synthesize current evidence on the link between IB dysfunction and depression. The focus is on biomarkers of intestinal permeability, in order to highlight potential pathophysiological mechanisms and their clinical relevance.

## 2. Materials and Methods

This narrative review is based on a structured search of the PubMed and Embase databases, aiming to identify studies published from 2015 to 2025 that examined the association between depression and blood-based markers of intestinal permeability. The search focused on key biomarkers including zonulin, intestinal fatty acid-binding protein (I-FABP), lipopolysaccharides (LPS), LPS-binding protein (LBP), and soluble CD14 (sCD14), which reflect distinct mechanisms of IB dysfunction. The selection of these biomarkers was made prior to the literature search. We predefined the set of biomarkers based on their ability to capture complementary aspects of intestinal barrier dysfunction, including tight junction regulation, enterocyte damage, microbial translocation, and immune activation. An important consideration was their feasibility, as all can be reliably measured in peripheral blood, providing a minimally invasive and clinically relevant approach, particularly suitable for psychiatric populations. Search terms included the following: “depression”, “intestinal permeability”, “intestinal barrier”, “biomarkers”, “zonulin”, “intestinal fatty acid binding protein”, “I-FABP”, “lipopolysaccharide”, “LPS”, “endotoxins”, “sCD14”, “lipopolysaccharide binding protein” and “LBP”.

**Figure 1:**
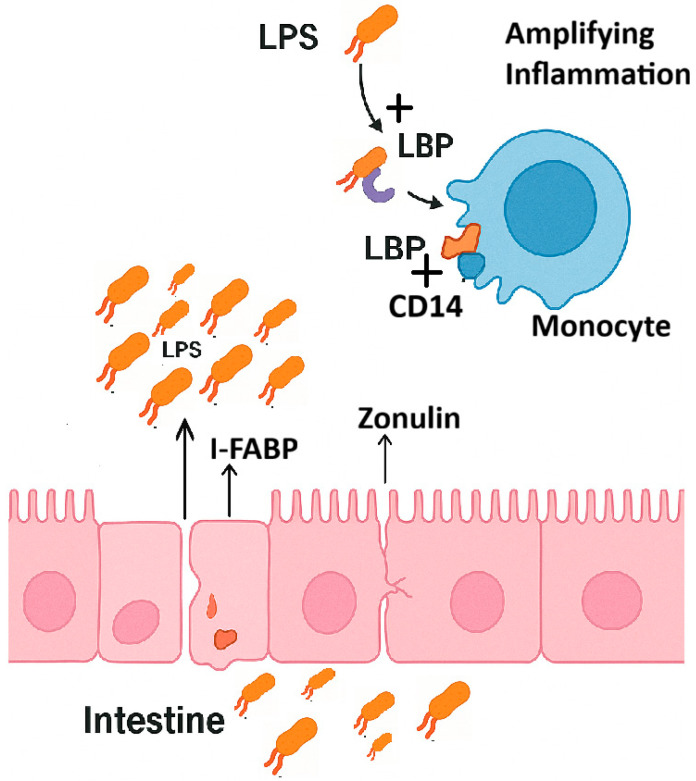
Mechanistic links between gut permeability markers and LPS-Induced immune activation. Abbreviations: **LPS** = lipopolysaccharides; **LBP** = LPS-binding protein; **CD14** = cluster of differentiation 14; **I-FABP** = intestinal fatty acid-binding protein. This figure shows the mechanistic links between gut permeability markers and LPS-induced immune activation. Zonulin increases intestinal permeability by loosening tight junctions. Enterocyte damage is reflected by the release of I-FABP. These enable the translocation of LPS from the gut lumen into the bloodstream. In the systemic circulation, LPS binds to LBP, and the resulting LPS-LBP complex interacts with CD14 on monocytes, ultimately leading to inflammation.

Inclusion criteria were original English-language studies that assessed relevant biomarkers (zonulin, I-FABP, LPS, LBP, sCD14) in patients with depression or in conceptually informative preclinical models. Exclusion criteria included conference abstracts, editorials, commentaries, non-peer-reviewed sources, studies focusing exclusively on non-blood-based biomarkers (e.g., fecal or salivary), and studies lacking extractable data. Screening was conducted independently by two reviewers, with any discrepancies settled through discussion. Additional articles were identified through manual reference checking and studies older that 10 years were retained when conceptually or mechanistically informative. While this review is narrative in nature, relevant items from the PRISMA checklist were considered and incorporated where applicable to improve transparency of reporting. No formal bias assessment was performed. However, emphasis was placed on methodological robustness and control for key confounders. During manuscript preparation, the authors used ChatGPT-4 (OpenAI, 2025) to provide partial support in enhancing language clarity, for limited assistance with the formatting of Table 1 and to generate visual elements for Figure 1. All outputs were carefully reviewed and edited by the authors, who assume full responsibility for the final content.

An overview of the main intestinal permeability biomarkers identified is provided in Table 1.

The mechanisms linking intestinal permeability markers to inflammation are presented in a simplified manner in Figure 1.

## 3. Overview of Intestinal Permeability Markers

Methods for assessing intestinal permeability have evolved over time. Several techniques exist, including the administration of monosaccharides (e.g., mannitol), disaccharides (e.g., lactulose and sucralose), polyethylene glycol (PEG) 400 and ^51^Cr-EDTA, with the subsequent urinary measurement of the tracer molecules. These well-established tests are considered accurate but time-consuming, unstandardized, and constrained by uncertain reference values. Additionally, intestinal permeability can also be measured more directly, either by studying small tissue samples taken from biopsies or by using endoscopic techniques that allow the barrier function to be observed in real time [19]. Biomarkers commonly used to assess intestinal permeability include albumin, calprotectin, and zonulin, which are measured in stool samples, as well as the blood-based biomarkers I-FABP, LPS, LBP, sCD14 and zonulin [20,21]. Their reliability is subject to debate, as studies do not yet recognize these as official markers. Blood-based biomarkers are considered more practical because they can be measured from a simple blood draw, which is minimally invasive and generally well tolerated. In contrast, traditional permeability tests that require oral administration of sugars or other probes may involve lengthy collection protocols and greater patient burden. Stool-based markers can also be informative, but they are less acceptable to patients, while biopsies and endoscopic techniques are invasive and not feasible for large-scale or vulnerable populations. For these reasons, blood-based biomarkers are particularly suitable in psychiatric research, where simple, less invasive, and reproducible measures are preferable. A clarification is necessary to avoid the misinterpretation that there are specific IB permeability biomarkers for depression or psychiatric disorders. Rather, they reflect broader changes in gut barrier function and have been reported across a variety of disorders, including cardiac, hepatic, or metabolic conditions. In the context of depression, these markers may indicate a possible contribution of gut-related inflammation, though their role is likely to differ depending on the underlying clinical setting. Here, we discuss their limits and potential contributions in the assessment of IB dysfunction in depression.

### 3.1. Zonulin

Zonulin is the only known human protein capable of reversibly regulating intestinal permeability by altering tight junctions. It can be measured in blood, stool or intestinal tissue samples, and is considered one of the most reliable serum markers for “leaky gut syndrome”. In its intact single-chain form, it operates by activating the epidermal growth factor receptor (EGFR) through proteinase-activated receptor 2 (PAR2) [22]. Zonulin was identified in the year 2000 by the research group of Fasano as the human counterpart of the zonula occludens toxin elaborated by *Vibrio cholerae*, which reversibly induces tight junction disassembly [23]. Later, Tripathi et al. characterized zonulin as pre-haptoglobin 2, an inactive precursor [22]. The protein has a molecular weight of 47 kDa and the two most powerful triggers for its release are gluten and bacteria present in the intestine [23,24]. This indicates that zonulin could act as a mechanistic link between changes in the gut microbiota and IB function. Several Gram-negative bacteria (e.g., *Escherichia coli*, *Prevotella*, *Pseudomonas*, *Salmonella*) were linked to increased intestinal zonulin release and gut permeability. In contrast, certain Gram-positive bacteria (e.g., *Bifidobacterium*, *Lactobacillus*), considered protective of the IB integrity, were associated with lower zonulin levels [25]. Zonulin dysregulation results in the passage of environmental and bacterial antigens. This can precipitate the pathogenesis of several autoimmune disorders such as coeliac disease, in relation to which it was first identified. The observation of increased zonulin expression in the intestinal tissue during the acute phase of the disease, when the tight junctions are opened, proposed a causal role of this endogenous mediator [23]. The median value of zonulin is 34 ng/mL (±14 ng/mL) in healthy individuals [26].

### 3.2. I-FABP/FABP2

Fatty acid-binding protein (FABP) was first identified over 50 years ago as a cytoplasmic molecule that binds long-chain fatty acids, modulating their absorption [27]. Following the initial discovery, FAPB is now recognized as part of a family of nine proteins that weigh 14–15 kDa and are 126–134 amino acids in length. They are preferentially expressed in tissues where active lipid metabolism occurs. FABPs are named after their first isolation tissue [28]. The family includes liver (L-FABP), intestine (I-FABP), heart (H-FABP), adipose tissue (A-FABP), skin (E-FABP), ileum (Il-FABP), brain (B-FABP), myelin (M-FABP), and testis (T-FABP) proteins. FABPs are not uniquely specific to a particular cell type, and multiple FABP isoforms are expressed in most tissues. As a result of being the second classified isoform, I-FABP is also known as FABP2. It is expressed in the epithelium of the intestine throughout, most abundantly in the distal segment, but also in the liver [29]. Following enterocyte injury, I-FABP is released into circulation, its elevated levels serving as an indicator of intestinal epithelial damage [30]. Thus, I-FABP appears to be a possible serum biomarker for conditions associated with disruption in the IB integrity. Both I-FABP and L-FABP plasma levels were increased in intestinal diseases. In hepatocellular injury alone, only L-FABP was elevated [31]. Serum I-FABP concentrations in healthy subjects are reported to be low [32]. The threshold value for I-FAPB is generally set at 2 ng/mL [33].

### 3.3. LPS

An important consequence of IB dysfunction is the increased translocation of LPS to the systemic circulation. LPS are present on the surface of most Gram-negative bacteria and the host immune system responds drastically upon their detection. Thus, LPS are considered PAMPs, or pathogen-associated molecular patterns [34,35]. LPS play a crucial role in maintaining membrane integrity and facilitating bacterial interactions with external surfaces [36]. Their structural domains include lipid A, a hydrophobic anchor which aids in the stability of the outer membrane, the O-antigen, and the core oligosaccharide [34]. Westphal et al. established in the 1950s a hot phenol/water method for purifying LPS, which remains a standard today. Their research demonstrated that lipid A is responsible for the toxic effects of LPS [37,38]. For more than a century, endotoxins have been deliberately administered to humans for therapeutic purposes, the assessment of anti-inflammatory agents, and the investigation of fundamental aspects of endotoxin biology [38]. LPS administration results in a depression-like model in rodents that has been widely used to clarify inflammation-related depression mechanisms and treatment effects [14]. LPS stimulate monocytes and macrophages, with the consecutive release of inflammatory cytokines (e.g., IL-1, IL-6, TNF-α) and activation of mediators through signal amplification pathways [13]. Within an hour of intravenous LPS administration, two consistent physiological responses are present: fever and a rise in heart rate. Other symptoms, with variation between subjects, include chills, headache, muscle aches, nausea or photophobia. High LPS levels in the bloodstream increases the risk of multisystem organ failure and septic shock. Symptom resolution occurs in approximately 6 to 8 h. A typical stress response follows the injection of endotoxin, marked by an early ACTH surge and followed by a gradual cortisol rise over 3–4 h. Catecholamines (epinephrine and norepinephrine) peak within 1–2 h and decline over the next 4–6 h [39]. In addition to the systemic inflammatory response, LPS promote microglial activation and the chronic release of pro-inflammatory cytokines in the brain, generating neuroinflammation [14]. One of the most extensively investigated cytokines in this context is TNF-α, with increased levels in the hippocampus and frontal cortex at day 7 and onward following LPS administration. Behavioral assessments identified a persistent cognitive deficit, with severe impairments in responsiveness to the environment that may relate to diminished motivation or attentional dysfunctions [40]. Patients with depression also showed increased IgM and IgA responses to LPS, linked to enhanced immune-inflammatory activity and oxidative and nitrosative stress (O&NS) pathways [18]. Moreover, LPS increase blood–brain barrier (BBB) permeability. Upon examining samples from HIV-infected individuals, elevated plasma LPS, BBB disruption and neuroinflammation were identified. Cerebrospinal fluid (CSF) LPS were found undetectable in all samples. Plasma LPS levels correlated with inflammatory markers as well as with BBB permeability in HIV-infected subjects. These findings indicate that microbial translocation contributes to neuroinflammation and BBB disruption without direct entry into the central nervous system [41]. Thus, LPS can be considered potential biomarkers for mental disorders due to their role in triggering systemic inflammation and neuroinflammatory responses. Indicating bacterial translocation, LPS should not be significantly present in the blood in the case of an intact IB.

### 3.4. LBP and sCD14

Host response mechanisms to Gram-negative bacterial infections rely mainly on LPS recognition. The prevailing dogma until 1990 was that LPS activated immune cells via a non-specific mechanism [42]. This principle was finally disproved with the identification of LBP, first characterized by Tobias et al. (1986) as an acute-phase protein, part of the LPS recognition system [43]. Later Schumann et al. (1990) reported that LBP functions as a carrier protein for LPS in plasma, controlling LPS-dependent responses by forming high-affinity complexes that bind to monocytes and macrophages, which then secrete inflammatory cytokines [44]. Currently, host mechanisms that recognize LPS are known as some of the most sensitive. LBP, a protein with a molecular weight of approximately 60 kDa, is synthesized predominantly in the liver. It possesses opsonic activity, binding to the surface of bacteria and mediating the adhesion to macrophages, with consecutive phagocytosis [45]. However, LBP’s significance resides in the ability to enhance the association of LPS with CD14, in order to enable the LPS receptor, Toll-like receptor 4 (TLR4), activating the cell [46,47]. The key role of LBP in LPS-induced activation is evidenced by the markedly reduced LPS responsiveness in LBP-deficient mice [48]. Data shows that LBP is involved in a complex mechanism of immune regulation, which includes both up-regulation and down-regulation of inflammatory processes induced by LPS. LBP has a dual role in Gram-negative sepsis. LBP enhances mononuclear LPS-driven cell activation at low levels, but at higher concentrations, during early immune responses, it inhibits this activation [49]. TLR4 itself does not bind to LPS, it uses CD14 as a cofactor to present LPS to MD-2, which then modulates LPS recognition and interacts with TLR4 [50,51,52]. It can be alternatively stated that CD14 delivers LPS from LBP to the signaling receptor complex MD-2/TLR4. This interaction induces a fast response mediated by the MyD88-dependent signaling, which results in the activation of nuclear factor kappa beta (NF-κB) and MAP kinases, ultimately leading to the transcription of proinflammatory cytokines, including TNFα, IL-1β, and IL-6 [53,54]. TLR4 is internalized and induces a delayed response characterized by the activation of the TBK1–IKKε–IRF3 pathway for the synthesis of IFNβ [55]. This process generates a systemic inflammatory response resulting in tissue inflammation and perturbed homeostasis. Psychological stress increases the expression of TLRs on macrophages and amplifies NF-κB activation induced by LPS, through stimulation of the HPA and sympathetic nervous system (SNS) [5].

LBP transfers LPS to either membrane-bound CD14 (mCD14) or to a circulating, soluble CD14 form (sCD14), in order to create LBP/LPS/CD14 micelles [56]. sCD14 transfers monomeric LPS from LPS–LBP complexes to mCD14, activating the cell, or directly to the MD-2/TLR4 receptor complex on cells that do not express mCD14 [57,58]. Beyond monocytes and macrophages, CD14 was detected in many other cell types, including subsets of dendritic cells [59]. Many publications have recorded the importance of CD14 in LPS response. Haziot et al. (1996) found that CD14-deficient mice were resistant to lethal LPS administration, whereas control mice did not survive [60]. Serum CD14 levels are considered an indicator of the extent of endotoxin-induced cell activation, making it a potential marker of bacterial translocation. The increase in LBP is relatively slow compared to LPS or other acute phase reactants, making it an indicator of sustained interaction between bacteria and immune cells. Serum LBP levels are significantly increased in sepsis (approximately 46.4 μg/mL), compared with healthy subjects (approximately 5.7 μg/mL) [61].

## 4. IB Marker Alterations in Depression

Multiple lines of evidence support an association between systemic inflammation and depression. Prospective cohorts have reported that higher circulating IL-6 and TNF-α predict subsequent depressive symptoms, consistent with an inflammation-driven subtype of depression [62]. Clinically, a systematic review of 20 meta-analyses that evaluated anti-inflammatory agents in MDD suggested that adjunctive anti-inflammatory agents might reduce depressive severity. Celecoxib and Minocycline showed the most consistent superiority, while omega-3 fatty acids demonstrated smaller and less consistent benefits. Despite promising data, particularly for celecoxib, study quality was variable and heterogeneity was substantial in many analyses and does not yet justify routine clinical application [63]. Regarding symptom analysis, inflammation might relate more to atypical depression features (e.g., hypersomnia, hyperphagia, fatigue) than to the full syndrome, according to a 2023 systematic review [64]. This aligns with the notion of an inflammatory subset of depression. Experimental human endotoxemia from a randomized double-blind study induced transient increases in IL-6, IL-10, IL-1 receptor antagonist (IL-1ra) and TNF-α, alongside decreased mood and increased anxiety [65]. This provides mechanistic plausibility for immune signaling relevant to IB dysfunction and LPS translocation. Patients with MDD have also been reported to show elevated levels of LBP and I-FABP, alongside increased circulating inflammatory cytokines, including TNF-α and IL-1β, indicating a concurrent disruption of intestinal barrier integrity and systemic inflammation. Additionally, a greater CRP production in depression and marital distress was linked to higher LBP and LBP/sCD14 concentrations [66,67]. These findings support the pathophysiological pathway in which IB disruption, highlighted by enterocyte damage and bacterial translocation, may contribute to monocyte activation and a subsequent inflammatory state in depression. Similarly, the reported association between zonulin and IL-6/ IL-8 in acute stress, reinforces this link [68]. Still, causality between inflammation and depression remains debated. Age-stratified data and specific cohorts (e.g., late-life depression) sometimes show null associations between inflammatory cytokines and depression, underscoring heterogeneity [69,70]. Luning Prak et al. (2022) studied older adults with major depression while rigorously excluding comorbid inflammatory conditions. The study found no baseline differences in measured cytokines between depressed patients and healthy controls. Moreover, adding celecoxib to antidepressant therapy did not reduce cytokine levels or improve outcomes, challenging the notion that inflammation is an intrinsic driver of depression in all cases [69]. Human endotoxin models indicate that mood changes are short-lived and coincide with the acute inflammatory phase [71]. This argues against generalized, sustained causation in all patients. Taken together, such findings suggest that the inflammatory theory of depression is unlikely to apply universally but may be more relevant to specific subgroups of patients or to particular symptom dimensions rather than to MDD as a whole. This framing justifies our focus on IB markers as stratification tools to identify inflammation-related depressive phenotypes, while acknowledging inconsistent findings.

While intestinal permeability markers are frequently altered in individuals with depression, their correlation is not consistent across studies. There is ample evidence supporting associations between zonulin and mental illness, although findings remain partially inconclusive. While zonulin has yielded heterogeneous findings across depression studies, the overall trend indicates elevated levels in affected individuals, whereas serum FABP2 appears to show a more consistent increase [66,72,73]. A controlled experimental study in healthy male participants showed that serum zonulin levels increased within 10 min of acute stress exposure and decreased after 60 min. Highly stressed men also reported more abdominal symptoms [68]. These results are suggestive of transient stress-induced gut barrier disruption. However, its limited sample size and male-only design restrict the generalizability of the results. Elevated zonulin and FABP levels were also noted by Stevens et al. (2018) in individuals physically asymptomatic for gastrointestinal distress with a depressive or an anxiety disorder. Both correlated with plasma LPS and altered gut microbiome [73]. The inverse relationship between the two biomarkers was reported previously in a study on individuals with HIV, high I-FABP and low zonulin predicting mortality [74]. This discrepancy may stem from the fact that zonulin is a regulatory protein involved in tight junction modulation, rather than a marker of epithelial damage. Its levels are influenced by numerous factors, including circadian rhythm [75]. Moreover, inconsistencies in measurement methods, assay sensitivity and specificity, contribute to its variability. In contrast, FABP2 could offer a more stable indicator of IB dysfunction. While elevated zonulin may suggest increased gut permeability, lower levels do not necessarily indicate barrier integrity, highlighting the complexity of its interpretation. Some authors do not recommend one single zonulin measurement for the assessment of IB integrity. They propose the assessment of IgG and IgA antibodies against zonulin and other tight junction proteins, as antibodies were deemed more stable when measured [75] This conflicting data indicates the need for further research about the role of antibodies against tight junctions in patients with depression or inflammatory diseases. Some studies have reported no specific permeability marker alteration in depression. Maget et al. (2021) found no significant differences in serum zonulin levels between euthymic and depressed patients, nor between those taking antipsychotic or antidepressant treatment and patients without medication [76]. Iordache et al. (2022) reported no significant association between FABP2 or zonulin levels and depressive symptoms in individuals with inflammatory bowel disease [33]. In patients with depression, after a 28-day trial of probiotics, serum zonulin experienced no significant changes [77]. This data highlights once more the heterogeneity of responses associated with the disorder.

Elevated immunoglobulin levels against LPS have been documented in MDD as early as 2008. Serum IgM and IgA levels against enterobacterial LPS were significantly higher in patients with MDD compared to healthy controls [18,78]. Increased LPS concentrations in cases comorbid with depression were also identified in tissue samples from the oral cavity of patients with chronic apical periodontitis. Moreover, LPS levels showed positive associations with the severity of depression [79]. This finding is relevant because it supports the hypothesis that peripheral sources of inflammation, even originating outside the gut, may contribute to the pathophysiology of depression. Hostile marital interactions were associated with elevated LBP and higher LBP/sCD14 in individuals, especially in those with a mood disorder history [67]. Alvarez-Mon et al. (2021) also reported significantly elevated serum levels of LBP in depressive patients compared to healthy controls [72]. Unlike transient permeability markers such as LPS, elevated LBP levels reflect chronic exposure to bacterial endotoxins. In depression, where prolonged immune activation is frequently observed, this characteristic is extremely relevant. Zhong et al. (2022) conducted a case–control study including patients with post-stroke depression (PSD), post-stroke patients without depression, and healthy controls, aiming to explore the role of intestinal permeability and related inflammatory markers. While FABP2 was significantly elevated in PSD, sCD14 levels did not differ significantly among groups, indicating that bacterial translocation via CD14-mediated pathways was not a distinguishing feature of PSD in this cohort [80]. sCD14 is indicative of monocyte activation but is not necessarily linked to compromised gut integrity. This could explain the limited alterations in depressed patients [81]. Conversely, among HIV-positive individuals, greater somatic depressive symptoms were significantly associated with higher levels of monocyte activation, as indexed by sCD14 [82].

When examining major markers of intestinal permeability collectively, a 2025 systematic review and meta-analysis by Morena et al. synthesized evidence from 22 studies involving both adults and pediatric populations. The authors reported that individuals with depressive symptoms exhibited increased levels of I-FABP, zonulin, antibodies against bacterial endotoxins, and sCD14. In contrast, no consistent differences were found for LBP [83]. These findings highlight both the potential relevance and the heterogeneity of intestinal permeability biomarkers in depression, supporting their use as stratification tools rather than established diagnostic markers. Importantly, recent data suggest that the relationship with depression may not primarily involve LBP itself. Instead, it may be linked to the presence of autoantibodies against LBP. These antibodies were found to be significantly elevated in young psychiatric patients, particularly those with affective and anxiety disorders [84]. This observation raises the possibility that immunological responses targeting LBP could be more closely linked to psychiatric vulnerability than LBP concentrations alone. It also reinforces the importance of analyzing multiple biomarkers in parallel and adopting an integrative perspective. Antidepressive treatment such as selective serotonin reuptake inhibitors (SSRI) Paroxetine, tricyclic antidepressants Clomipramine and Amitriptyline and monoamine oxidase inhibitors (MAOI) Tranylcypromine can prevent LPS-generated microglial changes, microglial oxidative stress markers, or the production of inflammatory markers [85]. This data indicates that antidepressants might also act by altering the microglial phenotype, reducing neuroinflammation. In the VACS Biomarker Cohort, Stewart et al. (2020) found that antidepressant use was associated with lower levels of sCD14 among HIV-positive participants, suggesting a potential mitigating effect of treatment on monocyte activation [82]. Patients with higher baseline levels of I-FABP had better clinical outcomes after 6 weeks of SSRI treatment, as measured by the decrease in their depression score on the 24-item Hamilton Depression Rating Scale (dHDRS24) [86]. This may also suggest that individuals with greater IB damage respond better to treatments targeting gut-related inflammation. However, this interpretation should be approached with caution, as current evidence remains limited and further validation is needed.

Intestinal permeability markers are also frequently altered in conditions associated with depression, such as chronic immune-mediated inflammatory diseases (IMIDs), as well as in HIV and metabolic syndrome-related disorders. This is possibly due to the combined effects of sustained activation of immune pathways and chronic inflammation, alongside the psychological burden of the illness. Therefore, evaluating intestinal permeability markers in these disorders is important, as they may provide insight into shared inflammatory mechanisms contributing to depressive symptoms. Increased zonulin levels have been reported in individuals with ankylosing spondylitis and inflammatory bowel disease (IBS) [87,88]. A positive correlation between depressive symptoms and LBP was also highlighted in patients with IBS [33]. Zonulin dysfunction and elevated LPS levels were also repeatedly linked to conditions such as diabetes, obesity or cardiovascular disease [89,90,91,92]. Administration of *Bifidobacterium* supplements, together with a dietary fiber, to obese or overweight participants, resulted in changes in zonulin that significantly correlated with changes in trunk fat mass [93] LBP is linked to old age, obesity and metabolic syndrome, while sCD14 correlates with age but not with metabolic markers [94,95]. This lack of correlation may be due to the fact that sCD14 acts as a general marker of monocyte activation, influenced more by age-related immune changes than by metabolic dysregulation. FABP2 was linked to Type 1 Diabetes triggers, and, moreover, FABP2 gene variants may increase Type 2 Diabetes risk [96,97]. In patients with heart failure, I-FABP is associated with disease severity and low intestinal microbe diversity [98]. Probiotics and inulin combination ameliorated chronic inflammation and endotoxemia, as well as depression and anxiety levels in coronary artery disease patients [99]. Moderate to severe COVID-19 has been associated with lower levels of FABP2 in the bloodstream. As a result, FABP2 was proposed as a marker of infected enterocyte functional modifications [100]. COVID-19 is also a condition associated with higher rates of depression, possibly as a result of neuroinflammation and neurological complications, alongside social isolation and economic hardship [101]. Comparable to findings in other somatic disorders, the association between depression and COVID-19 may involve similar changes through shared inflammatory GBA mechanisms, driven by increased gut permeability.

## 5. Conclusions

Numerous studies highlight the role of intestinal permeability in stress and depression, though much remains to be understood. Prior research faced difficulties reaching clear conclusions due to differences in study design, variation in biomarkers, and the absence of standardized tests. In this review, only two databases (PubMed and Embase) were consulted, potentially introducing selection bias and overlooking relevant studies. The focus was also restricted to a limited number of blood-based markers, excluding stool or saliva biomarkers, and other emerging indicators of gut permeability. Included studies varied in methodology, biomarker assays, and population characteristics, limiting comparability. Confounding factors such as diet, medication use, and comorbidities were often insufficiently controlled. Nevertheless, reported alterations in IB permeability and immune activation markers suggest a potential role for bacterial translocation and low-grade systemic inflammation as mediating factors in the pathophysiology of depression. Investigating IB permeability beyond gastrointestinal conditions is a growing field. Modulating the gut microbiota and IB permeability may represent a promising, but still preliminary, therapeutic approach for mental disorders. To advance this field, future research should focus on longitudinal studies to assess temporal relationships between IB permeability markers and depressive symptoms, as well as randomized controlled trials testing microbiota-targeted interventions. The use of standardized biomarker assays and harmonized methodologies across cohorts could contribute to improving comparability and reproducibility. Establishing valid biomarkers and optimizing microbiota-based therapies will require integrating clinical data with mechanistic studies to clarify causal pathways and identify patient subgroups most likely to benefit. Overcoming such problems could contribute to the development of more effective psychiatric care, supporting a more holistic approach to mental health. Moreover, we anticipate that multimarker algorithms will outperform any single marker, as assessing several intestinal permeability biomarkers together could provide a more comprehensive picture of IB dysfunction in depression. Measuring a panel of markers rather than relying on one alone may help identify the specific pathways involved and better capture the heterogeneity of patients. The importance of IB markers in mental disorders lies in highlighting GBA involvement, identifying patient subgroups with phenotypes related to intestinal permeability and potentially guiding treatment stratification and personalized therapeutic approaches. By reflecting systemic immune activation and IB dysfunction, these biomarkers may serve as a valuable starting point for advancing our understanding of pathophysiological mechanisms.

**Key points**

Intestinal barrier dysfunction may contribute to the pathophysiology of depression through microbial translocation and low-grade systemic inflammation.

Blood-based biomarkers of intestinal permeability such as zonulin, I-FABP, LPS, LBP, and sCD14 have shown altered levels in patients with depression, although findings remain inconsistent.

Zonulin and FABP2 represent distinct dimensions of IB impairment, with FABP2 more consistently elevated across studies.

LPS-induced neuroinflammation and BBB disruption support its role in inflammation-driven depressive phenotypes.

Despite their potential, intestinal permeability biomarkers are not yet validated for clinical application in psychiatric practice.

## Figures and Tables

**Table 1:** Blood-Based Markers of Intestinal Permeability and Their Clinical Significance in the Context of Depression.

Marker	Role	What Modified Levels Indicate
**Zonulin**	Modulates tight junctions between intestinal cells	Increased intestinal permeability
**I-FABP**	Released upon intestinal epithelial cell injury	Enterocyte damage
**LPS**	Bacterial endotoxin from Gram-negative bacteria	Microbial translocation
**LBP**	Binds LPS and facilitates immune recognition	Increased LPS exposure
**sCD14**	Soluble receptor for LPS signaling	Immune activation

Abbreviations: I-FABP = Intestinal Fatty Acid-Binding Protein; LPS = Lipopolysaccharides; LBP = LPS-Binding Protein; sCD14 = Soluble Cluster of Differentiation 14.

## Data Availability

No new data were created in this study. All data discussed in this review are derived from previously published studies, which are cited in the reference list. Further inquiries can be directed to the corresponding author.
